# The Effect of an Educating versus Normalizing Approach on Treatment Motivation in Patients Presenting with Delusions: An Experimental Investigation with Analogue Patients

**DOI:** 10.1155/2013/261587

**Published:** 2013-10-23

**Authors:** Eva Lüllmann, Tania M. Lincoln

**Affiliations:** Section for Clinical Psychology and Psychotherapy, Department of Psychology, University of Hamburg, 20146 Hamburg, Germany

## Abstract

Until recently a widespread recommendation for clinicians was not to respond to the content of patients' delusions but to stress at an early time point that the patient has a mental illness (educating approach). An opposed recommendation is to validate the patients' symptoms and normalize them (normalizing approach). This study used an experimental design to compare the impact of these two approaches on treatment motivation (TM). A cover story about a person who develops persecutory delusions was used to guide a sample of 81 healthy participants who served as analogue patients into imagining experiencing delusions. This was followed by a random assignment to either an educating or a normalizing consultation with a fictive clinician. Consultations only differed in content. Finally, we assessed the participants' motivation to accept medication (Medication TM), psychological treatment (Psychological TM), and treatment offered by this particular clinician independent of the kind of treatment (Clinician-related TM). Participants in the normalizing condition showed higher Clinician-related and Psychological TM than those in the educating condition. Medication TM was unaffected by condition. Following our results using a normalizing approach seems to be advisable in a first-contact situation with patients with delusions and favourable to a simple educating approach.

## 1. Introduction

Communication with the patient is a central feature of mental health treatment. In treating delusions, the question of what constitutes a “good communication style” is controversial. There seems to be a considerable gap between patients' and clinicians' perspectives of good communication in the consultation. Many patients actively attempt to talk about their delusional beliefs [1] and expect the clinician to listen and respond to their problems [2].

This expectation stands in contrast to clinical practice. Through analysing conversations in routine psychiatrist-patient consultations, McCabe et al. [1] found that psychiatrists avoid responding to the patients' concerns and rather evade their questions. Van Meer [3] confirmed that many psychiatrists were traditionally trained not to respond to delusional beliefs. Although today the idea of discussing the content of patients' beliefs is somewhat more widespread, many clinicians still fear that responding to delusional beliefs in an empathic manner or discussing them will make them worse [4]. Consequently, clinicians try to communicate that the delusional belief is a symptom of a mental disorder. This so-called “doctor-knows-best” approach [4] aims to enhance insight into illness and in turn adherence. Many studies have demonstrated that insight predicts treatment success and treatment adherence (for an overview see [5]). The assumption that educating delusional patients about their mental disorder enhances adherence has, however, not been confirmed [6, 7]. 

Another communicational approach applied in clinical practice focuses on normalizing delusions. In this approach, the clinician responds to delusional beliefs by providing empathy and understanding for the behavioural and emotional responses to them and thereby validates these experiences. This attempt is derived from client-centred and cognitive-behavioural therapy of psychosis (CBTp) [8–10]. It serves to establish a good therapeutic relationship which has been found to be associated with adherence [11] and successful treatment [12, 13]. Furthermore, it serves to reduce catastrophic cognitions about “being mad,” which enhance stress and in turn can exacerbate psychotic symptoms [14]. 

 Evidently, clinicians are confronted with diverging recommendations about how to respond to patients with delusions. Should they educate the patient about the mental disorder in the hope of increasing insight and—as a consequence—adherence? Or should they normalize and validate symptoms in order to reduce distress and strengthen the therapeutic relationship? 

The present study uses an experimental design to investigate how each of the two communicational approaches outlined above impacts on a person's willingness to engage in treatment after a first contact with a mental health professional. Healthy participants served as analogue patients—a method that has been validated in other doctor-patient communication studies [15, 16]. We used role-played interactions as they have been found to have higher affective impact than video-taped interactions [17]. Participants were guided to imagine experiencing persecutory delusions and consulting a clinician. We manipulated two different consultation conditions. In the first condition, the clinician educated the participant about having a mental disorder. In the second condition, the clinician normalized and validated the patient's psychotic experiences. Participants were randomized to one of the conditions and were then compared in regard to different aspects of their potential treatment motivation. Treatment motivation served as a proxy for adherence that is an important predictor of outcome [11, 18] but could not be assessed directly in the “fictive” study design. Treatment motivation “plays a decisive role in the utilisation of psychotherapy” [19, page 378], and lack of treatment motivation is a common phenomenon among people with severe mental illness [20] and has been discussed to be associated with the failure to enter and comply with treatment as well as its success [21]. We assessed different aspects of treatment motivation such as the willingness to engage in medication treatment (medication treatment motivation) and psychological treatment (psychological treatment motivation) and to engage in any treatment offered by this particular clinician (clinician-related treatment motivation). 

The present study can be seen as a pilot study, as no study so far has used a comparable study design, and empirical data are lacking. We hypothesize that participants in the normalizing condition will report higher clinician-related treatment motivation due to the fact that they feel better understood by the therapist in this condition. Furthermore, we hypothesize that participants in the educating condition will report higher medication treatment motivation, as this condition directly focuses the participants' insight into the mental illness and in turn might enhance insight into the need for treatment. Both approaches are likely to also enhance psychological treatment motivation. 

## 2. Method

### 2.1. Overall Study Design

The main part of the study was designed as a randomized experimental group-comparison with two experimental groups. We used a block randomization to balance the number of participants across conditions. We added a within-subject design, in which the participants retrospectively compared the two experimental conditions and commented on them.

### 2.2. Participants and Setting

The experiment was conducted at the University of Marburg; all participants were students (*n* = 82). By participating, students were able to complete curriculum requirements. 

### 2.3. Procedure

**Figure 1:**
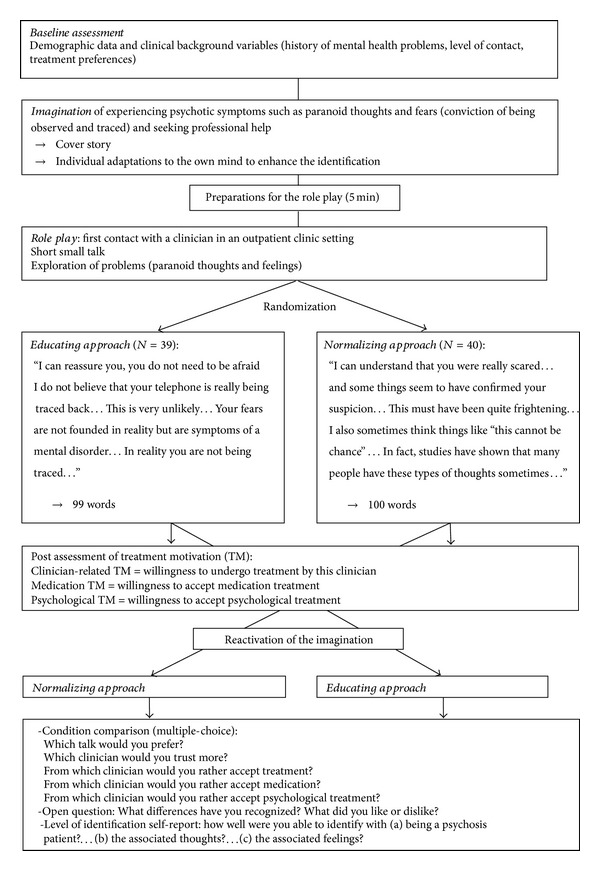
Design and course.

Figure 1 displays an overview over the course of the experiment.

A psychology student close to graduation conducted all experiments. The experiment took place in one-on-one encounters. First, participants were briefly informed about the scope and course. After consenting, participants provided information on demographic variables, previous mental health problems, treatment preferences for mental health problems, and previous contact with schizophrenia patients. Thereafter they participated in a guided imagination of experiencing paranoid thoughts and fears.

#### 2.3.1. Imagination

The experimenter read out an elaborated cover story about a person who develops the belief of being persecuted and of having his or her telephone and internet connections traced, finally seeking professional help on recommendation of good friends. Participants were asked to imagine being the protagonist and experiencing the plot themselves. 

Thereafter, the experimenter instructed participants to adapt the cover story to their own perspective by imagining circumstances under which they would become firmly convinced of being monitored and persecuted. In favour of a better identification, participants were allowed to change the cover story. The only defined part was the firm conviction of being monitored and having one's telephone and internet traced. Participants were instructed to imagine vividly how they would feel if they held such a belief. Finally, the participants were guided to imagine seeking help at an outpatient clinic and currently waiting for a clinician. To achieve a deeper identification with this situation, the experimenter left the room for five minutes. Participants were prepared to expect the beginning of a role-play when the experimenter reentered the room.

#### 2.3.2. Role-Play (Experimental Manipulation)

The role-play started with the alleged “clinician” (experimenter) entering the room dressed in a doctor's white coat. After a short small-talk the “clinician” explored the problems of the participant, that is, the paranoid belief, the circumstances of developing the belief, and the emotional responses. The exploration served to deepen the identification and to gain an impression of whether participants accurately imagined the defined delusional beliefs, which was an inclusion criterion for the analyses. During the exploration the “clinician” did not comment on the report of the participant. After the exploration the “clinician” responded with one of two experimentally manipulated and standardized statements (see also in Figure 1). 

In the educating condition, the “clinician” reassured the “patient” by stressing that the belief is not real and by underlining this position with rational arguments. The “clinician” did not go into greater detail concerning the delusional belief or the “patient's” reactions to it. Instead, she educated the “patient” about having symptoms of a mental illness and that this illness is causing the paranoid thoughts and fears. 

In the normalizing condition, the “clinician” validated the delusional belief by providing empathy and understanding for the feelings and reactions to it. The “clinician” underlined the normalizing approach by referring to own temporary feelings of being observed in everyday life and to studies about similar thoughts in the general population.

Both statements ended with an offer to provide professional help in coping with the problem.

The experimenter presented both statements in free speech in a calm and friendly tone. All role-plays were tape-recorded. A postgraduate clinical psychologist extensively trained and supervised the experimenter to administer the standardized intervention correctly and realistically and provided feedback in regard to wording and style based on listening to the first tapes. Additionally, the tapes were used to control for biases in the presentation of statements. We randomly picked out 15 tapes from each condition and presented them to two independent raters who were uninformed about the topic of the study and who were instructed to rate all tapes on 5-point Likert-scales with regard to friendliness and adequacy of speech rate. Raters were explicitly requested to disregard the content. The interrater reliability was very good, with Cohen's kappa = 85.72% for speech tone and 90.76% for speech rate. Independent *t*-tests showed no significant differences between the conditions (*t* = −0.185, *df* = 28, and *P* = 0.855 for speech tone; *t* = −0.285, *df* = 28, and *P* = 0.778 for speech rate).

After the role-play the participants completed questionnaires about their willingness to (a) accept treatment offered by this clinician (disregarding the type of treatment), (b) to accept medication treatment, and (c) to accept psychological treatment.

**Figure 2:**
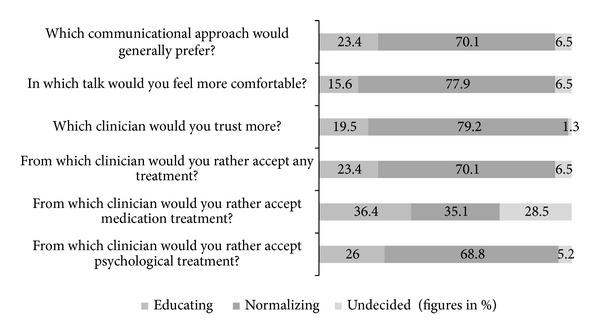
Results of the multiple choice condition comparison.

Thereafter, participants were instructed to re-imagine their personal cover story and were prepared to take part in another role-play. In this part (Part 2) the experimenter presented the respective other communicational approach. Participants were then asked multiple choice questions about their preferences between the two conditions, for example, in which conversation they would have more trust in the clinician and in which condition they would be more motivated to undergo the different types of treatment (“first version,” “second version,” and “undecided”). The items are displayed in Figure 2. Participants were also able to comment on the approaches and specify what they found helpful or less helpful. Finally, participants had to rate how well they were able to identify with (a) being a patient with delusions and (b) with the associated thoughts and (c) feelings during the experiment. To be included in the study participants had to be able to identify at least somewhat with being a patient (maximum score of 4 on a 5-point Likert-Scale ranging from 1 (very well) to 5 (not at all)). All of the participants fulfilled this criterion. We calculated mean scores for the three items to be able to control for differences in level of identification in the further analyses.

### 2.4. Questionnaires

Treatment motivation (TM) was assessed with a questionnaire that captures three dimensions of TM [22]. (1) Clinician-related TM (willingness to accept treatment offered by this particular clinician independent of the type of treatment): this dimension includes six items concerning the belief that treatment from this clinician will be helpful and the willingness to engage in a treatment from this clinician even if side effects occur or if considerable effort is necessary. (2) Medication TM (willingness to accept medication): this dimension uses the same item structure as the first dimension, but items refer to the belief that medication will be helpful and so forth. (3) Psychological TM (willingness to accept psychological treatment): again, the item structure remains the same, but items assess the belief that psychological treatment will be helpful and so forth. All items are rated on a 5-point Likert-Scale resulting in a minimal score of 6 and a maximum score of 30 per dimension. Psychometric properties of the questionnaire are good. The three dimensions were confirmed in a principal component analysis (PCA) factors were found to be highly consistent (Clinician-related TM: *α* = 0.90, Medication TM: *α* = 0.85, and Psychological TM: *α* = 0.89; [22]). 

The participants' previous experiences with persons with schizophrenia were assessed with the level of contact report [23]. The questionnaire assesses the extent of contact to people with schizophrenia, for example, whether they have relatives with schizophrenia or work with people with schizophrenia.

### 2.5. Statistical Analysis

Data were analyzed with PASW Statistics 18. To identify possible confounding variables we tested for associations between demographic variables, level of contact and level of identification, and the dependent variables (TM) using Pearson and Spearman correlations and for differences between experimental groups in these variables using *t*- and chi-square tests. We used multivariate analysis of variance (MANOVA) to examine the effect of experimental conditions on the combined dimensions of TM. We used *t*-tests for independent samples to locate statistically significant differences and calculated Cohen's *d* as an indicator of effect sizes.

We used the Kolmogorov-Smirnov Test to test for normality, which was only significant for age and level of contact. We used Spearman correlations to test for associations with these variables.

## 3. Results

### 3.1. Preliminary Analyses

#### 3.1.1. Success of Cover Story and Exclusion of Participants

The exploration during the role-play revealed that one participant had imagined depressive symptoms (lack of initiative, depressed mood) and two participants had imagined social phobic symptoms (fear that others might dislike them) rather than delusional beliefs. These three participants were excluded from the analyses, leaving a final sample of *n* = 79. Of these 69% were students of psychology, 31% studied other subjects. The mean age was 21.2 years (SD = 2, range 19–28); 86% of participants were female. 

The mean level of self-reported identification with the protagonist after the role-play was 2.19 (SD = 0.72) indicating that participants were able to identify with being a psychosis patient reasonably well. No participant reported a complete failure to identify.

#### 3.1.2. Test for Baseline-Differences and Potentially Confounding Factors

Experimental groups did not differ in age (*P* = 0.121), sex (*P* = 0.780), subject of study (*P* = 0.257), own experiences with psychological problems (*P* = 0.711), treatment of psychological problems (*P* = 0.498), treatment preferences (*P* = 0.572), or level of contact (*P* = 0.844). They also did not differ significantly in level of identification (*P* = 0.550) with the protagonist.

None of the potentially confounding variables (age, sex, study subject, own experiences, treatment preferences, and level of contact, level of identification) was significantly associated with TM.

### 3.2. Part 1: Test of the Effect of Communicational Approach on TM

There was a significant effect of communicational approach on the combined TM = 0.12  (*F*(3,74) = 3.3; *P* = 0.025, *η* ^2^ = 0.12). Results of *t*-tests are displayed in Table 1. *t*-tests revealed significant effects for Clinician-related TM and for Psychological TM, indicating that participants in the normalizing condition reported significantly higher Clinician-related and Psychological TM. Calculations of Cohen's *d* revealed a medium effect size for these two dimensions of TM. Medication TM did not differ between conditions.

Results remained unchanged after repeating the analysis including the three persons that had not met the inclusion criteria.

### 3.3. Part 2: Retrospective Comparisons of Interventions

Figure 2 displays the results of the multiple choice questions comparing the two experimental interventions. (For two participants the answers to the open questions indicated that they had compared the interventions with the exploration part rather than with each other. We therefore excluded these two data sets from this analysis.) Seventy to eighty percent of the participants felt more comfortable, had more trust in the clinician, and were more motivated to undergo treatment in the normalizing compared to the educating condition. Neither approach was considered as advantageous in regard to the motivation to undergo medical treatment.

To analyze participants' comments, we reviewed all comments and derived categories to summarize the content. A lay rater then assigned the content of comments to the categories. As most comments included more than one aspect, comments could be included in several categories.  Table 2 displays all categories and the frequency of comments that were included in the category per condition and in total.

Comments on the educating condition were most frequently (*n* = 28) categorized as “invalidating/offending/denying.” Comments on the normalizing condition were most frequently (*n* = 49) categorized as “understanding/empathic/validating.” 

## 4. Discussion

This study investigated the impact of an educating versus normalizing communicational approach on treatment motivation in an initial consultation with a “patient” with delusions in an analogue patient sample. Overall, the normalizing approach was more successful in motivating the participants to take up (any kind of) treatment with this clinician as well as to undergo psychological treatment. The motivation to take medication was unaffected by the communicational approach.

Our results contrast the traditional view that responding to paranoid beliefs and normalizing them only “makes it worse” and that clinicians should educate patients from the beginning about the delusional nature of their beliefs in order to enhance insight and treatment motivation. On the contrary, in our study the normalizing approach resulted in higher overall treatment motivation. Even in motivating participants to take medication the educating approach was not superior. According to the comments made, many participants felt invalidated and offended by the educating approach, which might have caused reactance thereby producing reduced overall treatment motivation. The normalizing approach resulted in higher clinician-related and psychological treatment motivation. The majority of participants felt more comfortable and validated and had more trust in the clinician in this condition indicating a better “therapeutic relationship” which might have been the major mediator of the positive effect of the normalizing approach on treatment motivation. This is in line with research that shows a good therapeutic relationship to be a predictor of treatment adherence [24, 25]. However, comments provided by seven participants indicated that they felt confirmed in their paranoid thoughts by the clinician in the normalizing condition. Even if this was only stated by a small number, it must be noted that the normalizing approach bears the risk of being misinterpreted. However, feeling believed should not necessarily lead to a stronger conviction that the delusion is real. Therefore, this risk seems to be less detrimental than the risk of invalidating or even offending a patient which seems to be a danger of the educating approach. 

One inherent element of the normalizing approach is to provide empathy and understanding for thoughts and feelings. However, empathy is not necessarily unique to the normalizing approach and could be used in educating approaches. We made sure that both conditions were comparable with regard to the warmth and friendliness of the speech tone. However, the benefit of the normalizing approach might be partly explicable by the empathy provided in this condition, and it would be interesting to test whether an educating approach that places more emphasize on empathy would produce similar effects.

Furthermore, in the present study we artificially contrasted educating and normalizing approaches and reduced them to their core aspects. The educating approach intentionally focused on the traditional psychiatric approach in which discussing psychotic symptoms is regarded as useless or even harmful [14] and in which psychotic symptoms are seen as not amenable to reason [26]. It is still present among some psychiatrists [27] and is likely to impede the clinician from being truly empathic. Our findings, however, can be generalized only to this type of simple educating approaches and not necessarily to the more complex psychoeducational approaches with interactive or coping-oriented elements [7]. Also, in clinical practice, the approaches need not be mutually exclusive. Psychoeducational approaches could be provided with more empathy, and a normalizing approach can be imbedded into a broader therapeutic approach that includes psychoeducational elements, such as educating the patient about the underlying mechanisms of delusions in Cognitive Behavioral Therapy for psychosis. In this context the normalizing approach may also facilitate the acceptance of the diagnosis as it helps to decatastrophize the term “schizophrenia” or “psychosis” and may help to take the patient “on board” to find alternative explanations for their symptoms [14] and in this way may help to enhance insight. 

### 4.1. Strengths and Limitations

To our knowledge, this is the first study that used an experimental design to investigate the impact of communicational approaches in mental health settings. As already applied in other doctor-patient communication studies [16, 17] we used analogue patients. However, imagining the experience will never capture the full experience and distress. Patients with clinical delusions, often accompanied by cognitive dysfunctions, might respond differently and benefit from direct and clear instructions. Nevertheless, the validity of using healthy samples seems justified by research showing that delusional beliefs lie on a continuum to normal experiences and are common in the general population [28]. We chose healthy participants attempting to create a fictive first episode setting without biases caused by previous experiences. Furthermore—considering ethics—a study that manipulates communication approaches in real patient contact should be preceded by a pilot study in a healthy population in order to assess benefits and dangers of the two approaches. We chose students as they have been shown to be particularly delusion prone compared to the general population [29]. Additionally, the mean age in student samples corresponds to the typical age of onset in psychosis. However, the educational level of our sample was not representative of patients with delusions. Possibly, persons with a lower educational level might feel more comfortable with a clinician who calms them and clearly points out that the experiences are not real. Although the majority of participants were psychology students which may have contributed to the overall high psychological treatment motivation we did not find differences in the different aspects of treatment motivation between psychology students and other students. Furthermore, the high psychological treatment motivation is in line with results about public attitudes toward psychiatric treatment [30, 31].

We used a personalized imagination in order to achieve a deeper identification with the experience of delusions. In spite of controlling for level and accuracy of imagination, the individual adaption of the cover story might have produced variations between participants. Another problem was that three participants misunderstood instructions and imagined having other symptoms and another two misunderstood instructions for the second part of the experiment. We avoided extensive instructions in the role-play to keep participants as focused as possible on the identification, which seems to have increased the likelihood of misinterpretations.

Another limitation is that we did not directly assess possible mediators for the impact of the approaches, for example, insight or the full concept of the therapeutic relationship. The reason for not including these variables was to have a fluent course of the experiment with as little interruptions of the sequences as possible in order to keep participants in the mental scene. However, we extracted indicators of the therapeutic relationship from the comments and the multiple choice questions.

### 4.2. Implications and Outlook

Our findings demonstrate that normalizing is a promising communicational approach for persons presenting with delusions in a first-contact setting. Simply educating the patient about the mental disorder turned out to be less helpful. However, we need more research in patients with clinical delusions in order to draw final conclusions about the benefit of the different communicational approaches. Furthermore, future studies should also investigate possible mediators such as insight or the therapeutic relationship.

Our study represents an important step forward in research in this field as it does not support the long-held hypothesis that normalizing approaches “make delusions worse.” On the contrary, they seem to be helpful in regard to treatment motivation and, possibly, adherence. Therefore, clinicians might be advised to replace the educating approach and concentrate on the relationship by normalizing symptoms, providing empathy, and understanding. Our results corroborate existing treatment recommendations such as those provided by Riecher-Rössler et al. [32], who recommend to build up trust, listen to the patient, recognize the patients' (psychotic) fears, and respect the patients' view of his or her problems. Vauth and Stieglitz [33] point to the necessity of validating the emotional consequences of the delusional belief. Helpful recommendations for responding to patients' delusions are also provided by Amador in his “Listen-Emphasize-Agree-Partner” (LEAP) Program [4].

## Figures and Tables

**Table 1:** Condition comparison of treatment motivation (independent *t*-tests).

	Educating	Normalizing	*t* (77)	*P* (2-tailed)	Cohen's *d *
	M (SD)				
Clinician-related treatment motivation	20.54 (3.95)	22.65 (3.46)	2.53	.013	0.568
Medication treatment motivation	16.74 (5.28)	16.08 (4.45)	-0.61	.544	0.135
Psychological treatment motivation	21.31 (5.34)	23.68 (3.84)	2.27	.026	0.509

**Table 2:** Analysis of the content of the open questions.

Categorized comments	Frequencies		Total
	Educating	Normalizing	
Understanding/empathic/validating	2	49	51
Invalidating/offending/denying	28	0	28
Emphasizes the illness	20	0	20
Calming	5	4	9
Normalizing/feeling of not being alone with the problem	0	9	9
Professional, serious, rational	8	0	8
Confirms paranoid thoughts	0	7	7
Makes me feel like an idiot/crazy	6	0	6
Personal, confidential	0	5	5
Convincing	1	3	4
Disturbing	1	1	2
Giving hope	1	1	2
Unprofessional	0	1	1
Caused paranoid thoughts about the clinician	1	0	1
Unconvincing	1	0	1
